# Increased insulin receptor binding and increased IGF-1 receptor binding are linked with increased growth of L6hIR cell xenografts *in vivo*

**DOI:** 10.1038/s41598-020-64318-4

**Published:** 2020-04-29

**Authors:** Henning Hvid, Tine Glendorf, Jakob Brandt, Rita Slaaby, Anne Lützen, Kim Kristensen, Bo F. Hansen

**Affiliations:** 1 Global Drug Discovery, Novo Nordisk A/S, Copenhagen, Denmark; 2 Global Research Technologies, Novo Nordisk A/S, Copenhagen, Denmark

**Keywords:** Endocrine system and metabolic diseases, Drug development, Preclinical research

## Abstract

Insulin analogue X10 has a higher mitogenic potency than native human insulin *in vitro* and supra-pharmacological doses of insulin X10 increased the incidence of mammary tumours in rats. Compared to native human insulin, insulin X10 has increased binding affinity to the insulin receptor and the IGF-1 receptor, but it is not known whether either or both characteristics are important for stimulation of cell proliferation *in vivo*. The aim of this study was to explore how increased binding affinity to the insulin receptor or the IGF-1 receptor contributes to stimulation of cell proliferation *in vivo*. A mouse xenograft model was established with rat L6 myoblast cells transfected with the human insulin receptor (L6hIR cells) and effects of supra-pharmacological doses of native human insulin, insulin X10 or novel insulin analogues with increased binding affinity to either the insulin receptor or the IGF-1 receptor were examined. Treatment with insulin X10 and insulin analogues with increased binding affinity to either the insulin receptor or the IGF-1 receptor increased growth of L6hIR cell xenografts significantly compared to native human insulin. Thus, increased binding affinity to the insulin receptor and the IGF-1 receptor are each independently linked to increased growth of L6hIR cell xenografts *in vivo*.

## Introduction

In the fast-acting insulin analogue X10 (IX10) histidine is replaced with aspartic acid at position B10. More than two decades ago, clinical development of IX10 was discontinued because supra-pharmacological doses of IX10 increased the incidence of mammary tumours in female rats in a chronic toxicity study^1^. The human relevance of this finding is unknown, but IX10 is still considered a “super-mitogenic” insulin analogue, and during non-clinical development of novel insulin analogues it is recommended to characterize the mitogenic potency *in vitro* and the carcinogenic potential *in vivo*^2^. Compared to human insulin (HI), IX10 binds with increased affinity to the insulin receptor (IR) and the IGF-1 receptor (IGF-1R) and has delayed dissociation rate upon binding to the IR^3–5^. However, it is not known if each of these characteristics independently are involved in enhanced stimulation of growth *in vivo*, even though these characteristics in the case with IX10 all appear linked with an increased tumour incidence in rats. In previous studies it has been reported that HI and certain insulin analogues stimulated growth of tumour cells *in vivo* only via binding to the IR^6–9^, but more recently it was demonstrated that HI and IX10 are in fact able to activate the IGF-1R on cancer cells *in vivo*^10^. However, in that study neither HI or IX10 stimulated the growth of the cancer cell xenografts, and it was therefore not possible to conclude whether increased binding affinity to the IGF-1R correlated with increased growth or not.

For the design and development of novel insulin analogues, it will be a clear advantage to fully understand which of the characteristics of IX10 can lead to increased stimulation of growth *in vivo*. The aim of the present study was therefore to examine how increased binding affinity to the IR and the IGF-1R contribute to stimulation of growth *in vivo*. To do this, the effect of repeated treatment with HI and IX10 was characterized in a xenograft model with rat L6 myoblast cells transfected with human IR (isoform A). Furthermore, the effect of treatment with two novel insulin analogues, designed to have increased binding affinity to either the IR or the IGF-1R, was examined, to establish how these characteristics influence cellular growth *in vivo*.

## Results

### Insulin analogues with increased binding affinity to either the insulin- or IGF-1 receptor display increased mitogenic potency *in vitro*

Relative to HI IX10 had ≈2.5-fold increased binding affinity to the IR and ≈5-fold increased binding to the IGF-1R (Table 1). This correlated with ≈2-, ≈4- and ≈6-fold increased mitogenic potencies in L6hIR, H4IIE and COLO-205 cells, respectively (Table 1). Analogue A showed ≈4-fold increased IR binding affinity and ≈5-fold lower IGF-1R binding affinity compared to HI (Table 1). This correlated with increased mitogenic potency in H4IIE cells (Table 1), where the mitogenic response is mediated via the IR (Supplementary Table S1). The binding affinity of analogue B to IR was comparable to HI, but the IGF-1R binding affinity was ≈25-fold increased (Table 1). In agreement with this, analogue B displayed increased mitogenic potency in COLO-205 cells, where the mitogenic response is driven primarily via the IGF-1R (Supplementary Table S1). Interestingly, neither analogue A or B displayed increased mitogenic potency in L6hIR cells *in vitro* (Table 1), despite the fact that this cell line expresses ≈287,000 IRs and ≈26,000 IGF-1Rs per cell (Supplementary Table S1).

**Table 1:** Relative receptor binding affinities and mitogenic potencies in L6hIR, H4IIE and COLO-205 cells.

Compound	Mutations	hIR-A binding affinity (% of HI)	hIGF-1R binding affinity (% of HI)	mitogenic potency L6hIR cells (% of HI)	mitogenic potency H4IIE cells (% of HI)	Relative mitogenic potency COLO- 205 cells (% of HI)
HI	None	100	100	100	100	100
IX10	B10Asp	251 [231; 272]	492 [472; 514]	204 [155; 269]	409 [347; 481]	640 [472–870]*
Analogue A	A8Arg, B26Glu, B28Glu, desB30	398 [375; 422]	18 [17; 19]	76 [60; 97]	158 [128; 196]	23 [18–30]
Analogue B	A21Arg, B10Glu, B29Arg, B31Arg, B32Pro, B33Lys	87 [80; 95]	2465 [2261; 2688]	83 [72; 96]	115 [93; 142]	4218 [3163–5625]

Data are weighted mean values with 95% confidence intervals from at least three independent experiments.

*As reported previously^10^.

### Insulin analogues can activate the insulin receptor and the IGF-1 receptor in L6hIR cells *in vitro* and *in vivo*

**Figure 1:**
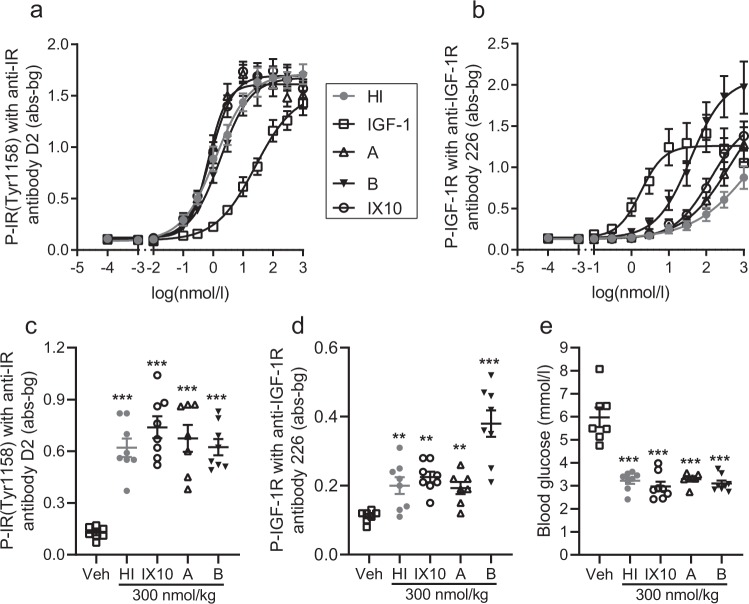
Activation of IR and IGF-1R in L6hIR cells *in vitro* and *in vivo*. **(a)** Activation of IR in L6hIR cells *in vitro* after treatment with HI (grey dots), IGF-1 (open squares), analogue A (open triangles), analogue B (black triangles) or IX10 (open circles). Symbols indicate mean values and error bars the SEM based on three replicates. The unit abs-bg is absorbance values with background values subtracted. **(b)** Activation of IGF-1R in L6hIR cells *in vitro* after treatment with HI, IGF-1, analogue A, analogue B or IX10. Symbols indicate mean values and error bars the SEM based on three replicates. The unit abs-bg is absorbance values with background values subtracted. **(c)** Activation of IR in L6hIR xenografts after treatment with vehicle or 300 nmol/kg HI, IX10, analogue A or B. Symbols indicate observations from individual animals and the horizontal line the mean ± SEM (*n* = 7 (vehicle and analogue A) or *n* = 8 (all other groups)). The unit abs-bg is absorbance values with background values subtracted. **(d)** Activation of IGF-1R in L6hIR xenografts after treatment with vehicle or 300 nmol/kg HI, IX10, analogue A or B. Symbols indicate observations from individual animals and the horizontal line the mean ± SEM (*n* = 7 (vehicle and analogue A) or *n* = 8 (all other groups)). The unit abs-bg is absorbance values with background values subtracted. **(e)** Lowering of blood glucose in mice with L6hIR xenografts after treatment with vehicle or 300 nmol/kg HI, IX10, analogue A or B. Symbols indicate observations from individual animals and the horizontal line the mean ± SEM (*n* = 7 (analogue A) or *n* = 8 (all other groups)). ***P < 0.001 vs the vehicle-treated group.

Treatment with HI, IX10, analogue A, B or human IGF-1 all resulted in acute activation of the IR and the IGF-1R in L6hIR cells *in vitro* (Fig. 1a,b) and the EC50 values for receptor activation were in good agreement with receptor-binding affinities (Supplementary Table S2). Treatment of mice with 300 nmol/kg HI, IX10, A or B, also resulted in acute activation of IR and IGF-1R in L6hIR xenografts *in vivo* (Fig. 1c,d), at comparable lowering of blood glucose (Fig. 1e).

### Supra-pharmacological doses of human insulin and insulin X10 have comparable pharmacokinetic properties

The PK properties of HI and IX10 at doses of 300 and 600 nmol/kg are shown in Supplementary Table S3. The maximum plasma concentration reached ≈500–850 nmol/l already at 11–17 min after s.c. injection and the elimination half-life of these doses equalled 1–1.5 h. There was a non-significant trend towards IX10 having a slightly longer half-life and mean residence time than HI, but the differences were only of 2–9 min. At the dose level of 300 nmol/kg IX10 displayed slightly faster clearance than HI, but at the higher dose level of 600 nmol/kg a non-significant trend in the opposite direction was observed. Overall, the PK characteristics of supra-pharmacological doses of HI and IX10 administered by s.c. injection therefore appeared to be comparable.

### Treatment with insulin X10 increases growth of L6hIR xenografts significantly compared to human insulin

**Figure 2:**
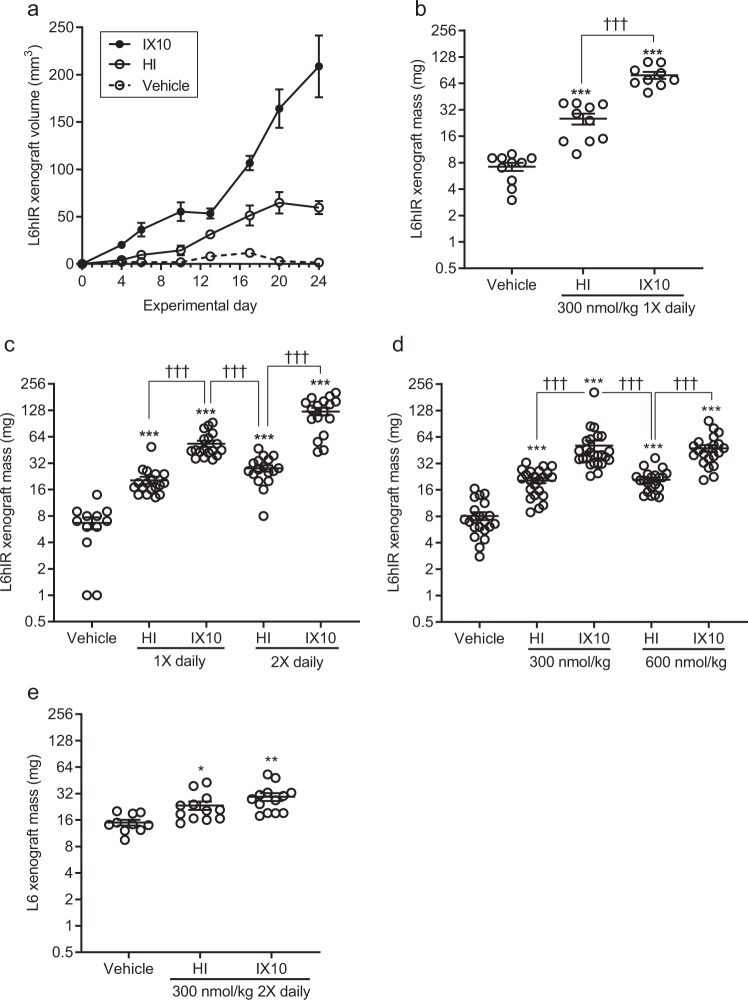
Effect of HI and IX10 on growth of L6hIR- and L6-xenografts. **(a)** Growth of L6hIR xenografts after treatment with vehicle (open circles), HI (open square) or IX10 (black dot) in experiment B. Symbols indicate mean values±SEM (*n* = 9 (IX10) or *n* = 10 (vehicle and HI)). **(b)** Mass of L6hIR xenografts at completion of experiment B. The y-axis is on logarithmic scale (log2). Symbols are observations from individual animals. Horizontal lines are mean ± SEM (vehicle: *n* = 10, HI: *n* = 10, IX10: *n* = 9). **(c)** Mass of L6hIR xenografts at completion of experiment C. The y-axis is on logarithmic scale (log2). Symbols are observations from individual animals. Horizontal lines are mean ± SEM (vehicle for 24 days: *n* = 12, HI 1X daily for 24 days: *n* = 18, IX10 1X daily for 24 days: *n* = 18, HI 2X daily for 21 days: *n* = 17, IX10 2X daily for 21 days: *n* = 17). **(d)** Mass of L6hIR xenografts at completion of experiment D. The y-axis is on logarithmic scale (log2). Symbols are observations from individual animals. Horizontal lines are mean ± SEM (vehicle: *n* = 21, 300 nmol/kg HI: *n* = 23, 300 nmol/kg IX10: *n* = 25, 600 nmol/kg HI: *n* = 21, 600 nmol/kg IX10: *n* = 21). **(e)** Mass of L6 xenografts at completion of experiment E. The y-axis is on logarithmic scale (log2). Symbols are observations from individual animals. Horizontal lines are mean ± SEM (vehicle: *n* = 10, HI: *n* = 12, IX10: *n* = 13). *P < 0.05, **P < 0.01 and ***P < 0.001 vs the vehicle-treated group. ^†††^P < 0.001 as shown.

Once daily treatment with 300 nmol/kg of HI or IX10 increased growth of the xenografts during the experimental period of 24 days (Fig. 2a), and the average mass of the xenografts was significantly increased in the groups treated with HI or IX10 compared to vehicle with ≈2.7- and ≈7.3-fold respectively (P < 0.0001, Fig. 2b and Table 2). Treatment with IX10 also increased xenograft mass significantly compared to HI with ≈2.7-fold (P < 0.0001 for both comparisons, Fig. 2b and Table 2).

**Table 2:** Mean ratios of xenograft mass after treatment with vehicle, HI or IX10.

Comparison	Mean ratio	95% confidence interval	P*-*value
300 nmol/kg 1X daily*			
HI vs. vehicle	2.7	[2.2; 3.4]	<0.0001
IX10 vs. vehicle	7.3	[6.0; 9.0]	<0.0001
IX10 vs. HI	2.7	[2.2; 3.3]	<0.0001
**300 nmol/kg 2X daily**			
L6hIR xenografts^†^			
HI vs. vehicle	4.9	[3.0; 8.1]	<0.0001
IX10 vs. vehicle	21.2	[12.8; 35.0]	<0.0001
IX10 vs. HI	4.3	[2.7; 6.8]	<0.0001
L6 xenografts^‡^			
HI vs. vehicle	1.5	[1.1; 2.1]	0.0121
IX10 vs. vehicle	1.9	[1.4; 2.6]	<0.0001
IX10 vs. HI	1.3	[0.9; 1.7]	0.1924
**600 nmol/kg 1X daily**^§^			
HI vs. vehicle	2.8	[2.0; 3.8]	<0.0001
IX10 vs. vehicle	6.1	[4.4; 8.3]	<0.0001
IX10 vs. HI	2.2	[1.6; 3.0]	<0.0001

*Combined analysis of groups from experiment B, C, D, F and G. Experiments are described in Table 4.

^†^Analysis of experiment C.

^‡^Analysis of experiment E.

^§^Analysis of experiment D.

With two daily treatments, the xenografts grew faster and in 21 days reached a larger average mass than seen with one daily treatment in 24 days (Fig. 2c). Treatment with HI 300 nmol/kg twice daily for 21 days increased xenograft mass with ≈4.9 fold compared to vehicle (P < 0.0001, Table 2), while treatment with IX10 300 nmol/kg twice daily for 21 days increased xenograft mass with ≈21.2-fold (P < 0.0001, Table 2). Twice daily treatment with IX10 further increased xenograft mass with ≈4.3-fold compared to HI, i.e., with two treatments per day the difference between HI and IX10 was larger.

Once daily treatment with 300 nmol/kg or 600 nmol/kg of HI or IX10 resulted in effects of comparable magnitudes (Fig. 2d and Table 2), in good agreement with the PK properties of these doses of HI and IX10. When the dose is doubled, the time with measurable plasma exposure is prolonged with one half-life. Since the half-life is only ≈1–1.5 h this means there was still no plasma exposure during the major part of the day.

As a further attempt to clarify how insulin and IX10 can stimulate growth, mass of the L6hIR xenografts was compared directly between (*i*) the group treated with 300 nmol/kg HI twice daily and the group treated with 300 nmol/kg IX10 once daily (Fig. 2c) and (*ii*) between the group treated with 600 nmol/kg HI once daily and the group treated with 300 nmol/kg IX10 once daily (Fig. 2d). In these comparisons the time with measurable plasma exposure, or the maximum plasma concentration, respectively, was up to twice as high for HI as for IX10. However, in both these comparisons, xenograft mass was significantly increased in the group treated with IX10 (P = 0.0009 and P < 0.0001, respectively, Fig. 2c,d). This strongly indicates that properties specific for the IX10 molecule is responsible for the increased stimulation of xenograft growth, and it can be excluded that the minimal differences in PK observed between IX10 and HI is responsible for the increased growth-stimulatory effects of IX10.

Finally, the effect of treatment twice daily with 300 nmol/kg HI or IX10 on growth of L6 xenografts (no IR overexpression) was explored (Fig. 2e). HI and IX10 increased L6 xenograft mass 1.5- to 2-fold (Table 2), but no significant difference was observed between HI and IX10. This emphasizes that the L6hIR xenograft model is a very sensitive model for detection of growth-stimulatory effects of insulin analogues.

### The stronger growth-promoting effect of insulin X10 on L6hIR xenografts is not mediated via increased gain of bodyweight or fat mass

**Figure 3:**
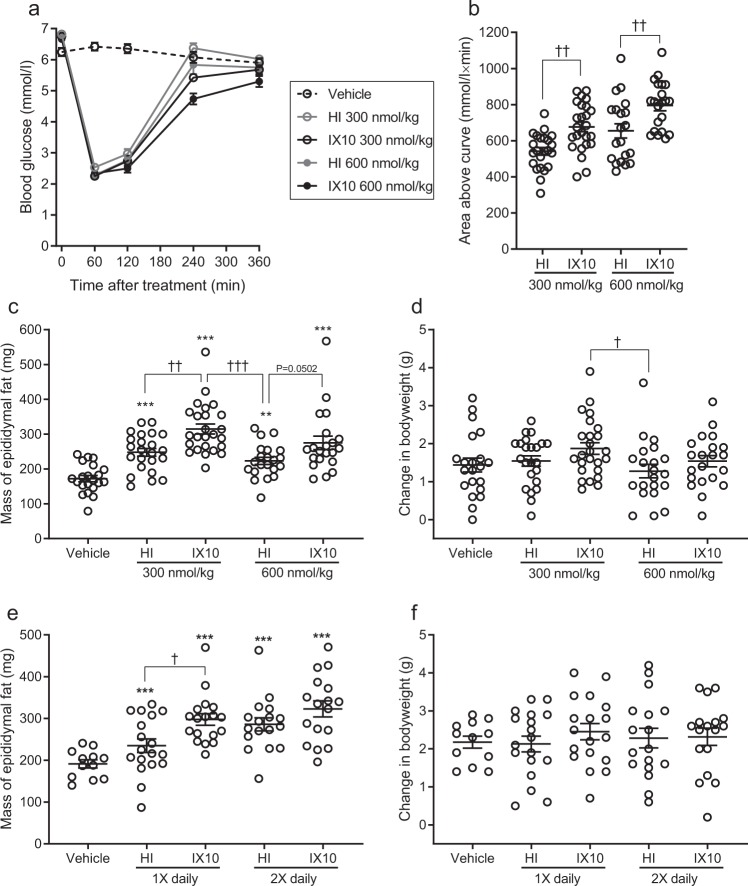
Metabolic effects of treatment with HI and IX10. **(a)** Mean blood glucose after treatment with vehicle (open triangle), 300 nmol/kg HI (open grey circle), 300 nmol/kg IX10 (open black circle), 600 nmol/kg HI (grey dot) or 600 nmol/kg IX10 (black dot). Symbols indicate mean values±SEM (vehicle: *n* = 21, 300 nmol/kg HI: *n* = 23, 300 nmol/kg IX10: *n* = 25, 600 nmol/kg HI: *n* = 21, 600 nmol/kg IX10: *n* = 21). **(b)** Area above the curve describing blood glucose 0–6 hours after treatment with 300 nmol/kg HI, 300 nmol/kg IX10, 600 nmol/kg HI or 600 nmol/kg IX10. Symbols are observations from individual animals. Horizontal lines are mean ± SEM (300 nmol/kg HI: *n* = 23, 300 nmol/kg IX10: *n* = 25, 600 nmol/kg HI: *n* = 21, 600 nmol/kg IX10: *n* = 21). **(c)** Mass of epididymal fat at termination of experiment D, after treatment with vehicle (*n* = 21), 300 nmol/kg HI (*n* = 23), 300 nmol/kg IX10 (*n* = 25), 600 nmol/kg HI (*n* = 21) or 600 nmol/kg IX10 (*n* = 21). Symbols are observations from individual animals. Horizontal lines represent mean ± SEM. **(d)** Change in bodyweight during experiment D, after treatment with vehicle (*n* = 21), 300 nmol/kg HI (*n* = 23), 300 nmol/kg IX10 (*n* = 25), 600 nmol/kg HI (*n* = 21) or 600 nmol/kg IX10 (*n* = 21). Symbols are observations from individual animals. Horizontal lines indicate mean ± SEM. **(e)** Mass of epididymal fat at termination of experiment C, after treatment with vehicle for 24 days (*n* = 12), 300 nmol/kg HI 1X daily for 24 days (*n* = 18), 300 nmol/kg IX10 1X daily for 24 days (*n* = 18), 300 nmol/kg HI 2X daily for 21 days (*n* = 17) or 300 nmol/kg IX10 2X daily for 21 days (*n* = 17). Symbols are observations from individual animals. Horizontal lines indicate mean ± SEM. **(f)** Change in bodyweight during experiment C, after treatment with vehicle for 24 days (*n* = 12), 300 nmol/kg HI 1X daily for 24 days (*n* = 18), 300 nmol/kg IX10 1X daily for 24 days (*n* = 18), 300 nmol/kg HI 2X daily for 21 days (*n* = 17) or 300 nmol/kg IX10 2X daily for 21 days (*n* = 17). Symbols are observations from individual animals. Horizontal lines are mean ± SEM. ***P < 0.001 vs the vehicle-treated group. ^†^P < 0.05, ^††^P < 0.01 and ^†††^P < 0.001 as shown.

Treatment with 300 or 600 nmol/kg of HI or IX10 decreased blood glucose in the mice for 4–6 h, and at each dose level, treatment with IX10 resulted in ≈20% larger area over the curve describing blood glucose (Fig. 3a,b). This means that a supra-pharmacological dose of X10 resulted in a longer-lasting decrease of blood glucose than treatment with an equimolar dose of HI. Treatment with HI or IX10 also increased mass of the epididymal fat compared to vehicle (Fig. 3c). In good agreement with the enhanced effect of IX10 on blood glucose lowering, treatment with 300 nmol/kg IX10 increased mass of the epididymal fat significantly more than HI (P = 0.0024, Fig. 3c) and comparable non-significant trends were seen when animals were treated with 600 nmol/kg once daily (Fig. 3c) or with 300 nmol/kg twice daily (Fig. 3e). However, these effects on an adipose tissue depot did not result in a significantly larger gain of bodyweight during the study period (Fig. 3d,f). To examine if the effects of HI and IX10 on growth of L6hIR xenografts were mediated via these metabolic effects, the variables epididymal fat mass and bodyweight gain were included in the statistical model of xenograft mass in addition to the variable treatment (mediation analysis principle, see Supplementary Information and Supplementary Table S4). Change in bodyweight, but not epididymal fat mass, had a significant effect on xenograft mass (P = 0.0069), but the effect of treatment was still highly significant (P < 0.0001) and the differences between groups treated with vehicle, HI or IX10 were fully comparable to the effects observed when the effect of bodyweight was not included in the analysis (Supplementary Table S4).

### Insulin analogues with increased binding affinity to either the insulin or the IGF-1 receptor increase growth of L6hIR xenografts more than human insulin

**Figure 4:**
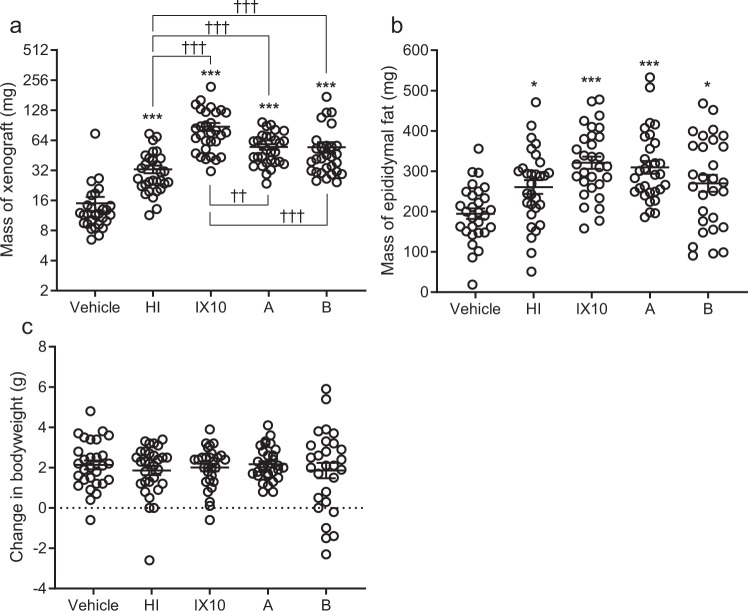
Effect of insulin analogue A and B on growth of L6hIR xenografts, bodyweight and epididymal fat. **(a)** Mass of L6hIR xenografts at termination of experiment F, after treatment with vehicle (*n* = 28) or 300 nmol/kg of HI (*n* = 30), IX10 (*n* = 30), analogue A (*n* = 30) or analogue B (*n* = 29). The y-axis is on logarithmic scale (log2). Symbols are observations from individual animals. Horizontal lines are mean ± SEM. **(b)** Mass of epididymal fat at termination of experiment F, after treatment with vehicle (*n* = 29), or 300 nmol/kg of HI (*n* = 30), IX10 (*n* = 31), analogue A (*n* = 30) or analogue B (*n* = 29). Symbols are observations from individual animals. Horizontal lines represent mean ± SEM. **(c)** Change in bodyweight during experiment F, after treatment with vehicle (*n* = 29), or 300 nmol/kg of HI (*n* = 30), IX10 (*n* = 31), analogue A (*n* = 30) or analogue B (*n* = 29). Symbols are observations from individual animals. Horizontal lines indicate mean ± SEM. *P < 0.05 and ***P < 0.001 vs the vehicle-treated group. ^††^P < 0.01 and ^†††^P < 0.001 as shown.

The effect of treatment with insulin analogue A or B on L6hIR xenograft growth is shown in Fig. 4a and Table 3. Mass of the xenografts was significantly increased after treatment with both analogue A (increased IR binding affinity) and analogue B (highly increased IGF-1R binding affinity) compared to the group treated with HI (P < 0.0001 and P = 0.0004, respectively, Fig. 4a and Table 3). Treatment with insulin analogue A and B also resulted in significantly smaller L6hIR xenografts compared to the IX10-treated group (P = 0.0025 and P < 0.0001, respectively, Fig. 4a and Table 3). Average mass of the epididymal fat depot was increased in all groups treated with HI or insulin analogues, and a non-significant trend towards a larger amount of epididymal fat in the groups treated with IX10 and analogue A was observed (Fig. 4b). The average change in bodyweight was not significantly different between any of the groups (Fig. 4c).

**Table 3:** Mean ratios of xenograft mass between treatment groups in experiment F*.

Comparison	Mean ratio	95% confidence interval	P*-*value
HI vs. vehicle	2.3	[1.7; 3.1]	<0.0001
IX10 vs. HI	2.6	[1.9; 3.5]	<0.0001
Analogue A vs. HI	1.7	[1.3; 2.3]	<0.0001
Analogue B vs. HI	1.6	[1.2; 2.1]	0.0004
IX10 vs. analogue A	1.5	[1.1; 2.0]	0.0025
IX10 vs. analogue B	1.6	[1.2; 2.2]	<0.0001

*Experiment is described in Table 4.

## Discussion

The principal findings in this study are that supra-pharmacological doses of IX10 increase growth of L6hIR xenografts significantly compared to HI, and that two model insulin analogues with increased binding affinity to either the IR or the IGF-1R also increase growth of L6hIR xenografts significantly compared to HI. Since the original report of increased incidence of mammary tumours in Sprague-Dawley rats treated with IX10^1^, this is the first study which report a significant and robust difference between IX10 and HI regarding stimulation of cellular growth *in vivo*. We furthermore demonstrate that IX10 and HI activate IRs and IGF-1Rs directly on L6hIR cells forming the xenografts, and that the increased growth-promoting effect of IX10 cannot be explained simply as an indirect effect of increased gain of adipose tissue or bodyweight. Finally, this study provides novel and important mechanistic insight, as it clearly demonstrates that increased binding to the IR as well as the IGF-1R independently correlate with an increased growth-promoting potential.

The L6 rat myoblast cell line is not derived from a cancer cell line and it can be argued that it is a poor model of potential spontaneous cancer cells in patients. However, the L6 and L6hIR cells are capable of unrestricted growth *in vivo* and therefore do resemble neoplastic cells to some extent. The L6hIR cells express ≈287,000 IRs per cell and ≈26,000 rat IGF-1Rs per cell. Most other cancer cell lines have at least 20-fold lower IR expression^11^. While human tumour tissues do express IR and IGF-1R^12–14^, the expression of IR in L6hIR cells is most likely considerably higher. It is therefore unlikely that spontaneous cancer cells will have a receptor expression profile like L6hIR cells. However, the primary aim of this study was not to test the effect of IX10 and HI in the most representative model of cancer in people with diabetes, but to explore the mechanisms which potentially increase the growth-potential of insulin analogues. Finally, it can be argued that the supra-pharmacological dose-levels used in this study have no clinical relevance. However, when possible growth-promoting effects of HI and insulin analogues are assessed in animal models, it is relevant to work with such high dose-levels, as discussed recently^10^.

Several previous studies have explored the effect of treatment with supra-pharmacological doses of HI and insulin analogues in various tumour models *in vivo*. It has been reported that IX10 increase growth of mouse colon tumour allografts, but no significant difference was found between groups treated with HI or IX10^15,16^, in agreement with the L6 xenograft data in this study. We also recently reported that neither HI nor IX10 increase growth of human colon cancer xenografts *in vivo*^10^. Another study reported that IX10 can increase growth of two mouse mammary tumour allografts but the effect of treatment with HI was not reported^6^. In a later study with the same allograft models, IX10 did not increase growth in all the reported experiments and HI had no effect compared to the vehicle-treated mice^7^. In a study with the p53^R270H/+^WAPCre mouse model of breast cancer, it was reported that chronic treatment with IX10, but not HI, significantly enhanced mammary tumour development and no significant difference was reported between the HI- and IX10-treated groups^17^. Gallagher *et al*.^6,7^ used doses of HI and IX10 which were four-fold lower than the maximum daily dose in the present study, and ter Braak *et al*.^17^ treated the mice with 12-fold higher doses of IX10 than HI, which complicates direct comparison. However, it is evident that the L6hIR xenograft model is a markedly different model. In the various models summarized above, the growth-promoting effect of IX10 is either absent or maximally increased with 1.3- to 2-fold compared to vehicle, and no robust difference has been found between HI and IX10. IX10 increases L6hIR xenograft mass ≈6- to 21-fold compared to vehicle, and ≈2- to 5-fold compared to treatment with HI, dependent on the experimental design (Table 2). These effects were consistently observed in five experiments. The L6hIR xenograft model may appear to be an overly-sensitive model, which allows for detection of growth-promoting signals from novel insulin analogues which have no relevance. However, the effect of a novel insulin analogue should always be compared to the effect of HI, and only effects stronger than HI should be considered a positive signal. When drug candidates are tested *in vivo* it is always a clear advantage to have sensitive models with a large difference between the effects of a positive and a negative control treatment. This is exactly the case for the effect of HI and IX10 in the L6hIR xenograft model.

Ever since the original finding of increased mammary tumour incidence in female rats treated with IX10 possible mechanisms have been discussed. Previously, it was suggested that increased IGF-1R binding was responsible^4^, whereas more recent studies argued that the increased growth-promoting effect of IX10 was caused solely by the increased binding affinity to the IR^6–8^. The current results demonstrate that insulin analogues with increased binding affinity to the IR as well as to the IGF-1R correlate with an increased mitogenic effect *in vivo*. This is in good agreement with previous *in vitro* studies with IX10^11,18^. Furthermore, this means that it is relevant to assess binding affinity to both the IR and the IGF-1R when novel insulin analogues are characterized *in vitro*. It also suggests that the stronger mitogenic effect of IX10 *in vitro* and *in vivo* is a result of the combination of increased binding affinity to both receptors. How increased binding affinity is connected to a stronger growth-promoting signal downstream of IR and IGF-1Rs, must be explored in future studies.

Dimerization of IR and IGF-1R occurs in the endoplasmatic reticulum. When IR and IGF-1Rs are expressed in the same cell, hybrid receptors will form by a random procedure and the relative amount of each receptor found as hybrid receptor can be calculated^19^. In L6hIR cells, the IR (isoform A) is expressed at ≈10-fold higher levels than IGF-1R (Supplementary Table S1), and therefore 90% of the IGF-1Rs can be calculated to exists as IR-A:IGF-1R hybrid receptors. However, relative to HI, the binding affinities of IX10 to IRs, IGF-1Rs, IR-A:IGF-1R and IR-B:IGF-1R hybrid receptors are in the same range^20^. It can be speculated that the correlation between IR:IGF-1R hybrid receptor binding and mitogenic effects/tumor growth promotion could be stronger than the correlation observed between IR or IGF-1R binding affinity and mitogenic effects. But it is not known whether an IR:IGF-1R hybrid receptor signals as an IR, an IGF-1R or displays hybrid-specific signalling. Because of the high IR expression relative to IGF-1R expression, 90% of the IRs in L6hIR cells will exist as IR homodimers. As demonstrated in this study, the L6hIR xenograft model therefore allows for detection of increased growth-promotion from insulin analogues with increased IR binding affinity as well as increased affinity to IGF-1R, which is found as a homodimer and in hybrid receptors in this cell line.

It is known that obesity correlates with an increased cancer incidence^21^ and that repeated treatment with insulin causes weight gain. This effect is also seen in the present study, where supra-pharmacological doses of IX10 resulted in a larger gain of adipose tissue compared to HI. One could therefore speculate if the increased mammary tumour incidence observed in rats treated for a year with IX10 was an indirect effect of increased weight gain. However, in the L6hIR model, the effect of IX10 was independent of increased gain of adipose tissue and bodyweight. We furthermore demonstrate that the increased growth seen after treatment with supra-pharmacological doses of IX10 cannot be explained by different PK properties and that treatment with HI or insulin analogues activates IRs and IGF-1Rs expressed on L6hIR-cells, i.e., the treatment-related effects on growth of L6hIR xenografts are direct effects on the cells forming the xenografts. This study is therefore in excellent agreement with *in vitro* mitogenicity studies and confirms that IX10 is a more mitogenic molecule than HI, and that IX10 also under *in vivo* conditions can stimulate cellular growth directly. This also implies that *in vitro* mitogenicity data generated across a panel of cell lines are predictive of growth-promoting effects *in vivo*, and therefore are relevant to assess during non-clinical characterization of novel insulin analogues.

In conclusion, IX10, which traditionally is considered a “super-mitogenic” insulin analogue, significantly increased growth of L6hIR xenograft tumours *in vivo* compared to HI. This is the first *in vivo* study which report a robust and significant difference in growth-promoting potential *in vivo* between HI and IX10 since the original report of increased mammary tumour incidence in female rats treated with IX10. Furthermore, increased binding affinity to the IR as well as the IGF-1R is linked to increased growth of L6hIR cell xenografts *in vivo*. Further studies are needed to elucidate how increased binding affinity contributes to a growth-promoting intracellular signal and what the nature of such a signal might be.

## Methods

### Synthesis of insulin analogues and assessment of insulin- and IGF-1 receptor binding affinity

Two novel insulin analogues were synthesized for this study. In analogue A, three amino acid substitutions (A8Arg, B26Glu and B28Glu), were introduced to decrease hIGF-1R affinity while retaining high hIR affinity. B30Thr is lacking (desB30) to allow for expression of the insulin analogue precursor in yeast and the subsequent enzymatic processing to remove the truncated C-peptide as described previously^22^. In analogue B, three amino acid substitutions were introduced (A21Arg, B10Glu and B29Arg) and three amino acids added to the native human insulin sequence (B31Arg, B32Pro and B33K) to increase hIGF-1R affinity (Table 1). Vector construction, precursor expression, conversion, and quantification of the insulin analogues were performed as described previously^23^. Receptor binding affinities were determined by competition assays essentially as described previously^24^.

### Cell culture, quantification of insulin- and IGF-1 receptor expression on COLO-205, L6hIR and H4IIE cells and assessment of mitogenic potency *in vitro*

Details of *in vitro* culture of COLO-205 cells, H4IIE cells, L6hIR cells and L6 cells (all mycoplasma free) are described in the Supplementary Information. Quantitative receptor expression and mitogenic potency of IX10 in COLO-205 cells have been reported previously^10,11^. Quantification of expression of IR and IGF-1R on L6, L6hIR and H4IIE cells was performed as described previously^11^, except that antibodies developed in-house which recognises rodent and human IGF-1R (anti IGF-1R antibody 226) and rodent and human IR (anti-IR antibody D2) were used. These two antibodies have been described previously^10^. The mitogenic potency of HI, IX10, analogue A and B was assessed from thymidine-incorporation as described previously^11^, except that 40,000 L6hIR cells and 60,000 H4IIE and COLO-205 cells were seeded per well.

### Animal experiments

This study included nine animal experiments, where different aspects of the effects of HI, IX10, analogue A or B on growth of L6hIR or L6 xenografts were explored. The aim of each animal experiment is shown in Table 4 and the detailed design of each experiment is described in the Supplementary Information and Supplementary Table S5. All experiments were performed in accordance with relevant guidelines and regulations. Furthermore, all experiments were approved by and performed under a license granted by the national Danish authority The Animal Experiment Inspectorate. Male BALB/c nude mice (CAnN.Cg-Foxn1 nu/Crl) were purchased from Charles River (Charles River Laboratories Germany, Sulzfeld, Germany) at age of seven weeks and housed five mice per cage in individually ventilated Techniplast GR 900 Seal Safe Plus cages, with unrestricted access to a complete pelleted rodent diet (Altromin 1324, Brogården, Hørsholm, Denmark) and tap water (non-chlorinated, non-acidified). In the animal room the temperature was 18–24 °C, with relative humidity at 30–70%, air change 8–15 times/h and a light-dark cycle of 12/12 h. All mice were acclimatized for 10 days before experiments were started. On day 0, all mice were injected s.c. in the right flank with 2.5 million L6hIR (six experiments) or L6 cells (one experiment), suspended in 0.1 ml PBS. On the same day treatment with vehicle or test compounds was started and continued either once or twice daily until the end of the experiment (Supplementary Table S5). All test compounds were produced at Novo Nordisk A/S and dissolved in a vehicle containing 5 mmol/l phosphate, 140 mmo/l sodium chloride and 70 ppm polysorbate 20 (Sigma-Aldrich, Brøndby, Denmark), and administered to the mice by s.c. injection in a dosing volume of ≈2 ml/kg, so the exact doses would be either 300 or 600 nmol/kg. Growth of the xenografts was monitored by measuring the length and width of each xenograft every second or third day during the experiment and calculating the xenograft volume according to the formula: volume = length × width^2^ × 0.52. All mice were weighed at the start, once weekly and at completion of an experiment. At the end of experiments mice were euthanized and the xenograft and the entire epididymal fat depot was dissected out of each mouse and weighed. Immediately thereafter the xenografts were snap-frozen in liquid nitrogen.

**Table 4:** Animal experiments included in this study.

Experiment	Purpose	Treatments included	Results described in
A	Acute IR and IGF-1R activation in L6hIR xenografts after a single acute treatment	Vehicle HI, 300 nmol/kg IX10, 300 nmol/kg Analogue A, 300 nmol/kg Analogue B, 300 nmol/kg	Fig. 1, Supplementary Table S5
B	Explore effect of treatment for 24 days on L6hIR xenograft growth	Vehicle HI, 300 nmol/kg 1X daily IX10, 300 nmol/kg 1X daily	Fig. 2, Table 2, Supplementary Table S5
C	Explore effect of treatment once daily (24 days) vs twice daily (21 days) on L6hIR xenograft growth	Vehicle HI, 300 nmol/kg 1X or 2X daily IX10, 300 nmol/kg 1X or 2X daily	Figs. 2, 3, Table 2, Supplementary Tables S4, S5
D	Explore effect of treatment with 300 vs 600 nmol/kg 1X daily for 24 days on xenograft growth	Vehicle HI, 300 nmol/kg or 600 nmol/kg 1X daily IX10, 300 nmol/kg or 600 nmol/kg 1X daily	Figs. 2, 3, Table 2, Supplementary Tables S4, S5
E	Explore effect of treatment with HI or IX10 twice daily for 24 days on L6 xenograft growth	Vehicle HI, 300 nmol/kg 2X daily IX10, 300 nmol/kg 2X daily	Fig. 2, Table 2, Supplementary Table S5
F	Explore effect of treatment once daily for 24 days with analogue A or B vs HI and IX10 on xenograft growth	Vehicle HI, 300 nmol/kg 1X daily IX10, 300 nmol/kg 1X daily Analogue A, 300 nmol/kg 1X daily Analogue B, 300 nmol/kg 1X daily	Fig. 4, Tables 2, 3, Supplementary Tables S4, S5
G	Explore effect of experimental treatments on L6hIR xenograft growth, treatment with HI and IX10 once daily for 24 days included as reference	Vehicle HI, 300 nmol/kg 1X daily IX10, 300 nmol/kg 1X daily	Table 2, Supplementary Tables S4, S5
H	Explore s.c. PK of 300 nmol/kg of HI and IX10	HI and IX10, 300 nmol/kg	Supplementary Tables S3, S5
I	Explore s.c. PK of 600 nmol/kg of HI and IX10	HI and IX10, 600 nmol/kg	Supplementary Tables S3, S5

### Assessment of blood glucose and plasma concentration of human insulin and insulin X10

Blood glucose and plasma concentrations of test compounds and endogenous mouse insulin were assessed as described previously^10^.

### Estimation of pharmacokinetic parameters of human insulin and insulin X10

Pharmacokinetic (PK) parameters were estimated from the two experiments with sparse sampling (three blood samples per mouse) for 0–240 min. Initially, population PK models^25^ for each compound was developed in Nonmem (Icon, Dublin, Ireland) and approximately 60 individual post-hoc concentration-time data points per mouse was subjected to non-compartment analysis.

### Assessment of activated insulin and IGF-1 receptor

L6hIR cells were seeded in 12-well plates and grown until 90–100% confluence in DMEM (Gibco, Invitrogen, Carlsbad, CA, USA). Cells were stimulated with increasing concentrations of ligands (0–1000 nM) for 30 min in DMEM (Gibco) medium containing 0.1% human serum albumin. Subsequently, cells were washed three times in ice-cold phosphate buffered saline (PBS) and snap-frozen by pouring liquid nitrogen into the wells. Cells were lysed in 100 µL lysis buffer (cell extraction buffer from BioSource, Invitrogen, Carlsbad, CA, USA; supplemented with 1 mmol/l 4-(2-aminoethyl)benzenesulfonyl fluoride hydrochloride, and protease inhibitor cocktail from Sigma-Aldrich). L6hIR cell xenograft samples were lysed in cell extraction buffer as described previously^10^. Protein concentration of cell and xenograft lysates was assessed with Pierce BCA protein assay kit (Thermo Fisher Scientific, Hvidovre, Denmark), according to the manufacturer’s instructions. Thereafter, levels of activated IR and IGF-1R were assessed in the lysates by sandwich ELISAs with the monoclonal human anti-IR D2 antibody and the monoclonal rabbit/human chimeric anti-IGF-1R 226 antibody as capture antibodies, as described previously^10^.

### Statistical analysis

Fitting of dose-response curves describing thymidine-incorporation *in vitro* and activation of IR and IGF-1R, including calculation of EC50 values, were conducted with GraphPad Prism (GraphPad Software Inc., La Jolla, CA, USA). Statistical analyses of data from animal experiments A-H were conducted with SAS JMP (SAS Institute Inc., Cary, NC, USA). For each experiment, each endpoint was analysed in a general linear model with treatment as explanatory variable. In experiments D, F and G the linear model also included the experimental block as an explanatory variable (see Supplementary Information for detailed description). Differences between groups were expressed as a ratio, and the analyses were therefore performed on logarithmically-transformed data (the natural logarithm) as described previously^26^. Tukey corrections was used as adjustment for multiple pairwise comparisons of groups in two-tailed t-test. Corrected P-values < 0.05 were considered statistically significant. The assumption behind the analyses of normal distribution was checked by evaluation of normal quantile plots and Shapiro-Wilks test for normality. The assumption of variance heterogeneity was checked by evaluation of plots of residual values against predicted values.

Treatment with 300 nmol/kg HI or IX10 once daily was included in experiment B, C, D, F and G and a combined analysis of L6hIR xenograft mass data from the groups treated with vehicle, HI or IX10 in these studies was performed as described above for each individual experiment, with treatment and study as explanatory variables.

To explore if the effect of HI or IX10 on xenograft mass was an indirect effect of the metabolic effects of HI and IX10, data for xenograft mass from experiment C, D, F and G were finally analysed as described above in a general linear model with treatment, study and either mass of epididymal fat or bodyweight gain (i.e., metabolic effects) included as explanatory variables. I.e., these analyses were based on the same principle as the mediation analyses described recently^27,28^ (see Supplementary Information for further description of this analysis).

## Supplementary information

Supplementary information.

## Data Availability

The datasets generated and analysed during the current study are available from the corresponding author upon reasonable request.
