# Supplementary material for: Increased insulin receptor binding and increased IGF-1 receptor binding are linked with increased growth of L6hIR cell xenografts in vivo
Source: Sci Rep. 2020 Apr 29;10:7247. doi: 10.1038/s41598-020-64318-4 (PMC7190841; doi:10.1038/s41598-020-64318-4)
Supplement: Supplementary file 1 — Supplementary information. [file 41598_2020_64318_MOESM1_ESM.docx]

**Title of manuscript:**

Increased insulin receptor binding and increased IGF-1 receptor binding are linked with increased growth of L6hIR cell xenografts *in vivo*

**Authors:**

Henning Hvid^1*^, Tine Glendorf^1^, Jakob Brandt^2^, Rita Slaaby^1^, Anne Lützen^1^, Kim Kristensen^1^, Bo F. Hansen^1^

**Affiliations:**

^1^Global Drug Discovery, Novo Nordisk A/S

^2^Global Research Technologies, Novo Nordisk A/S

**^*^Corresponding author:**

Henning Hvid

Global Drug Discovery, Novo Nordisk A/S,

telephone: +45 3075 4983

email: hhvd@novonordisk.com

ORCID iD: https://orcid.org/0000-0002-3667-4141

**Supplementary Information**

**Supplementary Methods**

***In vitro culture of cell lines used in the study***

COLO205 cells (ATCC, Manassas, VA, USA) were cultured in DMEM (Gibco, Invitrogen, Carlsbad, CA, USA) supplemented with 10% v/v FBS (Gibco), 100 µg/ml penicillin and 100 U/ml streptomycin (Gibco), 1% v/v non‐essential amino acids (Gibco) and 1% v/v Glutamax (Gibco). H4IIE cells (ATCC, Manassas, VA, USA) were cultured in minimum essential medium (MEM) (Gibco) supplemented with 10% (v/v) FBS, 100 µg/ml penicillin and 100 U/ml streptomycin, 1% (v/v) MEM non-essential amino acids and 1% (v/v) pyruvate (all from Gibco). L6hIR cells (generated at Novo Nordisk by stable transfection of L6 cells with human insulin receptor isoform A as described previously^1^) and L6 cells (ATCC, Manassas, VA, USA) were cultured in DMEM (Gibco) supplemented with 10% (v/v) FBS, 100 µg/ml penicillin and 100 U/ml streptomycin, and 1 mg/ml Geneticin (all from Gibco).

L6hIR cells and L6 cells for injection in mice were cultured as described above, and on the scheduled day for injection trypsinized, washed once in PBS, re-suspended in PBS at a concentration of 25 × 10^6^ cells/ml and kept on wet ice until injection.

***Detailed description of animal experiments***

See Supplementary Table S5 below for a detailed description of the design and number of animals included in each animal experiment.

***Animal randomization procedure***

In each experiment the animals were randomized to treatment groups using the following procedure:

- Each animal was assigned a random number using the RAND-function in Excel.
- The animals scheduled to be used in an experiment were sorted according to the size of the assigned random number, thereby generating a list with the animals in random order.
- On this list, the first animal was assigned to treatment group 1, the second animal was assigned to treatment group 2, the third animal was assigned to treatment group 3, etc., until the required number of animals was assigned to each treatment group.

In experiment D, F and G this randomization procedure was used within each experimental block.

***Blinding of animal experiments***

In experiment A the type of treatment of each animal was blinded for the laboratory technicians who performed the assays for activation of insulin receptor and IGF-1R.

In experiment B-G the type of treatment of each animal was blinded for the laboratory technicians who measured the size of the L6hIR xenografts in the animals and for the laboratory technicians who dissected and weighed the L6hIR xenografts and epididymal fat pads immediately after euthanasia of the animals.

In experiment H and I the type of treatment of each animal was not blinded for the laboratory technicians who analysed plasma samples for concentration of HI or IX10 (they needed to know which samples to analyse in which assays), but it was not possible for the laboratory technicians to influence the assay results or the PK-parameters derived from the non-compartment analysis.

***Determination of sample sizes***

In experiment A, the primary endpoint was assessment of activation of IR and IGF-1R in the L6hIR xenografts. Based on the variability in intracellular signalling in previous experiments with a comparable setup, we calculated that we with 7 animals per group were able to detect differences between two groups of minimum ≈50% (i.e., 1.5-fold), if such a difference existed. For the purpose of the study this was an acceptable minimum detectable difference, and we therefore included 7 or 8 animals in the groups.

In experiment B-G the primary endpoints were mass of the L6hIR xenografts at termination of the experiments. We based our sample size calculations on the effects and variability observed in experiment B, which was the first experiment we performed where mice with L6hIR xenografts were treated repeatedly with HI or IX10. In this experiment, HI increased mass of L6hIR xenografts with ≈3-fold relative to vehicle, and IX10 increased mass of L6hIR xenografts with ≈3-fold relative to HI. The coefficient of variation (CV) for xenograft mass in experiment B was ≈40%. The subsequent experiments were powered to allow for detection of effects of HI and IX10 which were also of smaller magnitude also in more variable conditions. For example, in experiment C we calculated that we with 18 animals per group were able to detect differences between HI and vehicle and between HI and IX10 (i.e., two parallel comparisons) down to ≈1.5 fold, even if the CV would be ≈50%. A difference of 1.5-fold would still be biologically relevant to detect. In the following experiment we included up to 31 animals per group, because we in some experiments planned multiple parallel pairwise comparisons, and because we learned that it was best to have a few extra mice included per group (i.e., 1-2 mice), in case some mice had to be euthanised prematurely (see also Supplementary Table S5).

In experiment H and I we included 15 animals per group and performed sampling of blood according to a sparse sampling scheme, see Supplementary Table S5. This number of animals was chosen because we by experience knew it would allow for sufficiently sensitive estimation of PK-parameters.

***Criteria for inclusion and exclusion of animals in statistical analyses***

Data from animals which completed the experiments and had developed a xenograft at euthanasia, and data from animals which were euthanised before planned termination (for various reasons, see Supplementary Table S5) and which had developed a xenograft at euthanasia were included in the statistical analysis. Data from the latter animals were included using the last observation carried forward-principle. Experiment B, C, D, F and G included in total 457 animals which received a s.c. injection with L6hIR cells and only 8 animals did not develop a xenograft. To obtain the most fair estimate of the effect of the various treatments on xenograft growth, these few animals must be excluded from the analyses, since no xenograft growth occurred at all in these animals. I.e., it was not possible to evaluate the effect of treatment in these very few animals.

***Statistical analysis of block experiments***

Experiment D, F and G were performed as block experiments, where the experimental blocks for practical reasons were staggered in time. In all experiments each group was represented in each experimental block. Statistical analysis of these experiments was done essentially as described for the other experiments, except that the variable experimental block and the possible interaction between treatment and experimental block was included as extra factors in the linear models. A significant interaction between treatment and experimental block was not detected in any of the analyses.

***Combined analysis of experiment B, C, D, F and G***

Treatment with vehicle, 300 nmol/kg HI 1X daily and 300 nmol/kg IX10 1X daily was included in experiment B, C, D, F and G. In order to obtain the most fair estimate of the effect of these treatments on growth of L6hIR xenografts, a combined analysis was performed. This was done by including treatment and study as explanatory variables in a general linear model. A significant interaction between study and treatment was not found. This combined analysis comprised 98 vehicle-treated animals, 108 animals treated with HI and 109 animals treated with IX10.

***Mediation analysis***

As described in the Results and Discussion sections the relatively stronger growth-promoting effect of IX10 could be speculated to be caused by a relatively stronger metabolic effect, e.g., enhanced lowering of blood glucose, increased gain of adipose tissue and increased gain of bodyweight when compared to treatment with an equimolar supra-pharmacological dose of HI. It was therefore relevant to explore to what extent the increased growth of L6hIR xenograft seen after treatment with IX10 could be explained by an enhanced metabolic effect, using the mediation analysis principle described previously^2,3^. In these analyses, we included data from the mice in experiment C, D, F and G, treated with either vehicle (*n*=88), 300 nmol/kg HI 1X daily (*n*=98) or 300 nmol/kg IX10 1X daily (*n*=100), because we in these experiments had assessed mass of the L6hIR xenografts as well as metabolic effects (mass of epididymal fat and change in bodyweight during the experiment) in all mice. The results of the analyses are described in Supplementary Table S4. All analyses were done with ln-transformed xenograft mass data, as described in the Methods section. First, we analysed only the direct effect of treatment, in a general linear model with treatment, study and the possible interaction between treatment and study as explanatory variables. F-tests were used to determine in factors had a significant effect or not. No significant interaction was observed, and treatment had a highly significant effect. Then we performed analyses where the direct effect of treatment and possible indirect effect of metabolic effects (i.e., epididymal fat mass or change in bodyweight, respectively) were included as explanatory variables in linear models together with study and the possible interaction between study and treatment. No significant interaction between study and treatment was found. In both analyses the direct effect of treatment was still highly significant. The variable mass of epididymal fat did not have a significant effect, and this possible indirect effect therefore did not explain the effect of IX10 on L6hIR xenograft mass. A significant effect of change of bodyweight was observed when this possible indirect effect was included as an explanatory variable (Supplementary Table S4). However, the direct effect of treatment was still highly significant, and when differences between the different treatment groups were estimated with the model that included both the direct effect of treatment and indirect effect of change in bodyweight, the differences were fully comparable to the effects observed when only the direct effect of treatment was used to model the effect of IX10 on L6hIR xenograft growth (See Supplementary Table S4). This means that the effect of IX10 on L6hIR xenograft mass only to a minimal extent could be explained by the relatively greater gain of bodyweight, and that the effect of IX10 by far can be explained by a direct treatment effect.

**Supplementary Table S1: Insulin and IGF-1 receptor expression quantified as average antibody binding sites per cell ± SD**

| **Cell line** | **Insulin receptors per cell** | **IGF-1 receptors per cell** |
| --- | --- | --- |
| L6 | 1,159 ± 778 (rat) | 17,067 ± 570 (rat) |
| L6hIR | 287,393 ± 121,693 (human and rat) | 25,894 ± 7,971 (rat) |
| H4IIE | 27,963 ± 5,354 (rat) | not detectable |
| COLO-205 | 2,123 ± 587 (human)† | 13,927 ± 1,346 (human)* |

*As reported previously^4^

**Supplementary Table S2: EC50 values and 95% confidence intervals for activation of IR and IGF-1R**

| **Treatment** | **P-IR(Tyr1158)** | | **P-IGF-1R** | |
| --- | --- | --- | --- | --- |
|  | EC50 (nmol/l) | 95% CI | EC50 (nmol/l) | 95% CI |
| HI | 1.2 | [0.8 ; 1.9] | 2,137 | [1,329 ; 4,412] |
| IX10 | 0.8 | [0.6 ; 1.1] | 275.4 | [196.7; 394.7] |
| Analogue A | 0.7 | [0.5 ; 0.9] | 498.2 | [356.3 ; 735.7] |
| Analogue B | 1.7 | [1.2 ; 2.6] | 34.6 | [22.2 ; 53.0] |
| IGF-1 | 25.8 | [14.2 ; 70.0] | 1.8 | [0.8 ; 3.7] |

**Supplementary Table S3: Pharmacokinetic parameters of supra-pharmacological doses of HI and IX10, presented as mean values and 95% CIs**

| **Treatment and dose** | **C_max_ (nmol/l)** | **t_max_ (min)*** | **t_1/2_ (min)** | **Clearance/F (ml/min/kg)** | | **MRT (min)** |
| --- | --- | --- | --- | --- | --- | --- |
| HI 300 nmol/kg | 725.7 [654.0 ; 797.4] | 11 | 96 [85 ; 107] | 13.2 [12.7 ; 13.7] | | 24 [22 ; 27] |
| IX10 300 nmol/kg | 502.3 [475.2 ; 529.6] | 14 | 105 [91 ; 119] | 17.0 [15.3 ; 18.7] | | 29 [27 ; 30] |
| HI 600 nmol/kg | 812.5 [761.7 ; 863.4] | 17 | 52 [48 ; 56] | 19.9 [17.9 ; 21.9] | | 31 [30 ; 32] |
| IX10 600 nmol/kg | 861.3 [816.5 ; 906.1] | 17 | 56 [49 ; 63] | 17.2 [15.3 ; 19.2] | | 33 [30 ; 35] |
| **Comparison of IX10 and HI** | **t_1/2_** | | **Clearance/F** | | **MRT** | |
|  | **mean difference (min)** | | **mean ratio** | | **mean difference (min)** | |
| IX10 vs HI, 300 nmol/kg | 9 [-8 ; 26] | | 1.27 [1.08 ; 1.49] | | 5 [1 ; 7] | |
| IX10 vs HI, 600 nmol/kg | 4 [-13 ; 21] | | 0.86 [0.73 ; 1.01] | | 2 [-1 ; 7] | |

*Median values

MRT=mean residence time

F=bioavailability

**Supplementary Table S4: Mediation analysis of xenograft growth***

| **Comparison** | **Analysis only of direct effect** | | **Analyses including direct and possible indirect effects** | | | |
| --- | --- | --- | --- | --- | --- | --- |
|  | Treatment (direct effect): P<0.0001 | | Treatment (direct effect): P<0.0001  Epididymal fat mass (indirect effect): P=0.7645 | | Treatment (direct effect): P<0.0001  Change in bodyweight (indirect effect): P=0.0069 | |
|  | mean ratio | 95% CI | mean ratio | 95% CI | mean ratio | 95% CI |
| HI vs. Vehicle | 2.7 | [2.3 ; 3.1] | 2.7 | [2.3 ; 3.1] | 2.7 | [2.3 ; 3.1] |
| IX10 vs. Vehicle | 7.2 | [6.2 ; 8.4] | 7.1 | [6.0 ; 8.5] | 7.1 | [6.1 ; 8.2] |
| IX10 vs. HI | 2.7 | [2.3 ; 3.1] | 2.7 | [2.3 ; 3.1] | 2.6 | [2.3 ; 3.0] |

*Analyses above are of the four studies where L6hIR xenograft mass and metabolic parameters were measured in the mice (i.e., experiment C, D, F, G)

**Supplementary Table S5: Design of animal experiments included in this study**

| **Experiment** | **Purpose** | **Details of experiment** |
| --- | --- | --- |
| A | Acute IR and IGF-1R activation in L6hIR xenografts after a single acute treatment | Duration: 21 days. All mice were treated with HI 300 nmol/kg by s.c. injection 1X daily on day 1-20 to stimulate growth of the L6hIR xenografts. On day 21, the mice were treated with either vehicle (n=7), HI 300 nmol/kg (n=8), IX10 300 nmol/kg (n=8), analogue A 300 nmol/kg(n=7) or analogue B 300 nmol/kg (n=8) by s.c. injection and euthanized 20 min later. |
| B | Effect of treatment on L6hIR xenograft growth | Duration: 24 days. The experiment included the following treatments: Vehicle (n=10), HI 300 nmo/kg 1X daily (n=10) or IX10 300 nmol/kg 1X daily (n=10), by s.c. injection. One mouse in the IX10-treated group did not develop a xenograft. |
| C | Explore effect of treatment once daily vs. twice daily on L6hIR xenograft growth | Duration: 24 / 21 days days. The experiment comprised the following treatments: Vehicle (n=12) for 24 days, HI 300 nmol/kg 1X daily for 24 days (n=18), HI 300 nmol/kg 2X daily for 21 days (n=18), IX10 300 nmol/kg 1X daily for 24 days (n=18), IX10 300 nmol/kg 2X daily for 21 days (n=18). All treatments were done by s.c. injection. One animal treated with HI 300 nmol/kg 2X daily and one animal treated with IX10 300 nmol/kg 2X daily was euthanized due to severe hypoglycaemia at experimental day 4. No xenografts were observed in these two mice were euthanized. |
| D | Explore effect of treatment with 300 vs. 600 nmol/kg on xenograft growth | Duration: 24 days. The experiment comprised the following treatments: Vehicle (n=21), HI 300 nmol/kg 1X daily (n=23), IX10 300 nmol/kg 1X daily (n=25), HI 600 nmol/kg 1X daily (n=21), IX10 600 nmol/kg 1X daily (n=21). All treatments were done by s.c. injection. The experiment was performed in three experimental blocks, each group was represented in each experimental block. |
| E | Explore effect of treatment with HI or IX10 twice daily on L6 xenograft growth | Duration: 24 days. The experiment comprised the following treatments: Vehicle (n=10), HI 300 nmol/kg 2X daily (n=12), IX10 300 nmol/kg 2X daily (n=13). All treatments were done by s.c. injection. |
| F | Explore effect of analogue A and B vs. HI and IX10 on xenograft growth | Duration: 24 days. The experiment comprised the following treatments: Vehicle (n=30), HI 300 nmol/kg 1X daily (n=30), IX10 300 nmol/kg 1X daily (n=31), analogue A 300 nmol/kg 1X daily (n=30), analogue B 300 nmol/kg 1X daily (n=30). All treatments were done by s.c. injection. The experiment was performed in three experimental blocks and all treatments were represented in each block. In the vehicle-treated group one mouse was euthanised prematurely because of bite-wounds and had not developed a xenograft. Another mouse from the vehicle-treated group had not developed a xenograft at termination. In the IX10-treated group 1 mouse had not developed a xenograft at termination. Another mouse from the IX10-treated group was euthanized prematurely (at day 22) because it had developed a very large xenograft. Data from this mouse were included in the analyses using the last-observation-carried-forward principle. Finally, 1 mouse in the group treated with analogue B was euthanized prematurely because of bite-wounds and had not developed a xenograft. |
| G | Explore effect of experimental treatments on L6hIR xenograft growth, treatment with HI and IX10 included as reference | Duration: 24 days. The following treatments were included: Vehicle (n=27), HI 300 nmol/kg 1X daily (n=27), IX10 300 nmol/kg 1X daily (n=28). All treatments were done by s.c. injection. The experiment was performed in two experimental blocks and all treatments were represented in each experimental block. One mouse in the IX10-treated group had not developed a xenograft at termination of the experiment. |
| H | Explore s.c. PK of 300 nmol/kg of HI and IX10 | Duration: 1 day (a single acute treatment to all animals). The experiment comprised the following treatments: HI 300 nmol/kg (n=15) and IX10 300 nmol/kg (n=15). The mice were treated by s.c. injection at time=0, and blood samples were collected 5, 10, 20, 30, 60, 90, 120, 180 and 240 min after treatment. Each mouse was only sampled at every third timepoint, i.e., for each treatment five mice were sampled at each timepoint. |
| I | Explore s.c. PK of 600 nmol/kg of HI and IX10 | Duration: 1 day (a single acute treatment to all animals). The experiment comprised the following treatments: HI 600 nmol/kg (n=15) and IX10 600 nmol/kg (n=15). The mice were treated by s.c. injection at time=0, and blood samples were collected 5, 10, 20, 30, 60, 90, 120, 180 and 240 min after treatment. Each mouse was only sampled at every third timepoint, i.e., for each treatment five mice were sampled at each timepoint. |

**Supplementary Information References**

1 Bonnesen, C. *et al.* Synchronization in G0/G1 enhances the mitogenic response of cells overexpressing the human insulin receptor A isoform to insulin. *Cell Biol Toxicol* **26**, 293-307, doi:10.1007/s10565-009-9142-x (2010).

2 Ahren, B. *et al.* Semaglutide induces weight loss in subjects with type 2 diabetes regardless of baseline BMI or gastrointestinal adverse events in the SUSTAIN 1 to 5 trials. *Diabetes Obes Metab* **20**, 2210-2219, doi:10.1111/dom.13353 (2018).

3 Vilsboll, T. *et al.* Semaglutide, reduction in glycated haemoglobin and the risk of diabetic retinopathy. *Diabetes Obes Metab* **20**, 889-897, doi:10.1111/dom.13172 (2018).

4 Lundby, A. *et al.* Surface-expressed insulin receptors as well as IGF-I receptors both contribute to the mitogenic effects of human insulin and its analogues. *J Appl Toxicol* **35**, 842-850, doi:10.1002/jat.3082 (2015).
